# Assessing utility values for treatment-related health states of acute myeloid leukemia in the United States

**DOI:** 10.1186/s12955-018-1013-9

**Published:** 2018-09-21

**Authors:** Eytan M. Stein, Min Yang, Annie Guerin, Wei Gao, Philip Galebach, Cheryl Q. Xiang, Subrata Bhattacharyya, Gaetano Bonifacio, George J. Joseph

**Affiliations:** 1 Memorial Sloan Kettering Cancer Center, New York, NY USA; 2 Analysis Group, Inc, Boston, MA USA; 3 Analysis Group, Inc, Montreal, QC Canada; 4 Novartis Healthcare Pvt, Ltd, Hyderabad, India; 5 Novartis Pharmaceuticals Corporation, East Hanover, NJ USA

**Keywords:** Acute myeloid leukemia, Discrete choice experiment, Health state, Preference valuation, Utility

## Abstract

**Background:**

Preference valuations of health status are essential in health technology and economic appraisal. This study estimated utilities for treatment-related health states of acute myeloid leukemia (AML) and disutilities of severe adverse events (SAEs) using a representative sample of adults from the general population in the United States (US).

**Methods:**

Treatment-related AML health states, defined based on literature and interviews with clinicians, included complete remission (CR), no CR, relapse, stem cell transplant (SCT), and post SCT short-term recovery. Six attributes with varying levels, including fever, lack of energy, problems with daily function, anxiety/depression, blood transfusions, and hospitalization, were used to define health states. An online survey using discrete choice experiment methodology was designed to capture preferences for health status scenarios including the identified attributes and key grade 3/4 chemotherapy-related SAEs. Health state utilities and SAE disutilities were generated from a conditional logistic regression with generalized estimating equations.

**Results:**

Of the 300 survey participants, the demographic distributions were within a 3% margin of those in the 2010 US Census. CR had the highest utility value (0.875), followed by post-SCT short-term recovery (0.398), relapse (0.355), no CR (0.262), and SCT (0.158). Of the SAEs, serious infection had the highest decline in utility (0.218), followed by severe diarrhea (0.176), abnormally low blood cell counts (0.100), and severe redness/skin peeling (0.060).

**Conclusions:**

AML and treatments can result in reduced quality of life and impaired ability to perform daily activities. Findings of this study underline the value that society places on treatment-related AML health states and SAEs.

## Background

Although a type of rare disease, acute myeloid leukemia (AML) is the most common form of acute leukemia in adults, which accounts for approximately 25% of all leukemia in adults in the Western world [1]. The median diagnosis age of AML is 68 years [2]. As the population ages, the incidence of AML is expected to rise. AML progresses rapidly if left untreated; even with treatment, the survivorship remains poor, particularly among a few subtypes of AML. The 5-year survival rate is low at approximately 27% [2]. In the United States (US), an estimated 21,380 new AML cases and 10,590 deaths related to AML were expected in 2017 [2, 3].

The diagnosis and treatment of AML can take a toll on a patient’s life. Studies have shown a substantial drop of health-related quality of life (HRQL) shortly after the AML diagnosis and persistent through the course of therapy [4, 5]. The current standard of care for AML consists of induction therapy to induce remission and post-remission consolidation chemotherapies. The treatment strategy for an individual is informed by a number of factors such as age, comorbidities, performance status, disease subtype [6]. Although many patients achieve complete remission (CR) from the initial induction chemotherapy, most of them relapse, with less than one-fifth maintaining CR after 1 year [2, 7, 8]. The intent of post-remission consolidation chemotherapies is to solidify CR and to delay relapse. However, adverse events are commonly associated with chemotherapies and can further compromise the HRQL of patients with AML. Eligible patients who achieve CR may receive allogeneic hematopoietic stem cell transplant (SCT), a potentially curative procedure, though relapse may still occur. Moreover, the process of SCT is logistically difficult (i.e., finding a donor match) and costly; it can substantially compromise patients’ HRQL due to the complexity of this invasive procedure, the associated intensive care for an extended hospitalization and the risks for adverse events (AEs) and complications [9]. A large HRQL decrement during the short-term SCT recovery period is expected within the first few months following the SCT procedure, though the majority of patients who survive the procedure gradually return to pre-procedure levels of HRQL within 1–4 years [9, 10].

New therapies are emerging to treat AML and to improve the HRQL of these patients. A proper assessment of utility values associated with health states of a disease and the treatment is important for the appraisal of new therapies during regulatory approval and for establishing their value for reimbursement [11]. Societal preferences are recommended for health economic evaluation in order to assess the optimal allocation of health resources among the general population [12, 13]. However, existing utility values are frequently lacking for rare diseases. AML disease specific utility values and those for toxicities associated with AML chemotherapies have yet to be established in the US [14].

Discrete choice experiment (DCE) is a well-established method for eliciting preferences for medical products or services in the healthcare field [15–19]. While standard gamble (SG) and time trade-off (TTO) are two conventional methods for estimating health state utility values, the DCE approach has been increasingly assessed for estimating health utilities [20–23]. However, previous studies using the DCE approach for utility assessment focused on the methodology feasibility itself and were based on EQ-5D, which is a generic utility assessment measure. The choice-based method has become one of the recommended methods for eliciting preferences [13], but the feasibility of using this approach to generate disease specific health state utilities is yet to be evaluated. This study applied the DCE method to estimate utilities from a US societal perspective for treatment-related health states, and dis-utilities of severe adverse events (SAEs) among patients newly diagnosed with AML who are fit for standard induction chemotherapy and for whom SCT is a potentially curative therapy. The health state utility values from this study can be used to generate the quality adjusted life years (QALYs) in cost-effectiveness/cost-utility analysis of a new AML intervention.

## Methods

With a DCE design, hypothetical scenarios of medical products, services, or health states of interest are defined by attributes and associated levels via surveys. Survey participants are presented with pairs of alternative hypothetical scenarios in the form of choice cards with competing levels of attributes. The responses to choice cards permit modeling of the probability of an alternative scenario being chosen as a function of the scenario attributes. Results from the model allow for an estimation of utilities based on the predefined health states. This study was designed with two steps: a qualitative phase to develop a descriptive system (i.e., health state classification) followed by a quantitative phase (i.e., a valuation study). The qualitative phase intended to identify the key attributes to define treatment-related AML health states and inform the important SAEs associated with chemotherapies that were impactful in the HRQL of these patients. A survey using the DCE approach was designed afterwards. In the quantitative phase, a valuation study was conducted in which the preference weights were obtained from the adult general population in the US. All procedures performed in the study involving human participants were in accordance with the ethical standards of the institutional and/or national research committee, and with the 1964 Declaration of Helsinki and its later amendments or comparable ethical standards. This study was granted an exemption status from the New England Institutional Review Board.

### Qualitative phase for descriptive system development

#### Identification of attributes and levels

A literature review and two rounds of in-depth interviews with experienced practicing hematology-oncology physicians and clinical experts were conducted. The literature review focused on AML disease course and efficacy criteria used in recent AML clinical trials to inform potential health states, and frequently reported SAEs associated with AML chemotherapies. With the goal of utility generation, the aspects that are impactful on AML patients’ lives were also reviewed to identify attributes that could be potentially used to define health states.

The literature search resulted in the development of initial discussion guides in the form of PowerPoint slides, which were used for one-on-one in-depth interviews with three clinical experts over the phone and WebEx was used to share slides to facilitate the discussions. Round 1 interviews focused on exploring the potential health states critical in managing patients after being diagnosed with AML and the most frequently reported SAEs associated with AML chemotherapies. Potential attributes and the associated levels were discussed for inclusion to define each of the health states. Clinicians were then asked to use layman language to describe the attributes as they would to patients in their clinical practice. In order to have the attributes/levels manageable for a DCE design, the clinicians were informed to limit the total number of attributes to no more than 12 and the levels for each of the attributes to no more than four. Feedback from Round 1 interviews were consolidated. A draft tutorial document in PowerPoint was developed to describe the attributes in layman language, which would be used to provide basic information for a participant to complete the survey. The AML treatment-related health states were defined by attributes with associated levels, and the SAEs for consideration were included. When discrepancies emerged during Round 1 interviews, these were highlighted for Round 2 discussions with those same clinicians from Round 1 interviews. Round 2 interviews were conducted 1 month after the completion of Round 1 interviews, and focused on confirming the health states definitions, resolving discrepancies, and reviewing the tutorial document. Each of the interviews lasted approximately 1 hour and Dr. Yang conducted all interviews.

As a result, five distinct AML treatment-related health states were identified: CR, no CR, relapse, SCT, and post-SCT short-term recovery (Table 1). These health states were predefined by six attributes typically encountered by AML patients, including fever, lack of energy, problems with daily function, anxiety/depression, blood transfusions, and hospitalization (Table 2). These attributes covered areas in patient-reported symptoms, function and mental health, and the care process, all of which were agreed to be important considerations when valuing quality of life of patients dealing with the disease itself and the associated treatments. In addition, four attributes were used to reflect four sets of Grade 3/4 SAEs (i.e., serious infection, severe diarrhea, severe redness/skin peeling, and abnormally low blood cell counts). Levels of the attributes generally referred to the presence/absence of the SAEs, frequency (e.g., rarely/never, occasionally, frequently), or severity of the attribute. The duration of life (1, 4, 7, and 10 years) was included as an attribute to articulate that the hypothetical health status scenarios reflected a life-threatening condition.

**Table 1:** List of AML treatment-related health states

Attribute	Complete Remission	No Complete Remission	Relapse	SCT event	Post-SCT short-term
	Feature value	Feature value	Feature value	Feature value	Feature value
Fever	Rarely/never	Frequently	Frequently	Frequently	Occasionally
Lack of energy	Occasionally	Frequently	Frequently	All the time	Frequently
Problems with daily function	Able to work normally	Not able to perform daily activities but able to self-care	Not able to work but able to perform daily activities	Not able to perform daily activities but able to self-care	Not able to work but able to perform daily activities
Anxious/depressed	Occasionally	Frequently	Frequently	Frequently	Occasionally
Blood transfusion	Rarely/never	Occasionally	Occasionally	Frequently	Rarely/never
Hospitalized	Rarely/never	Frequently	Frequently	All the time	Occasionally

Abbreviations: *AML* acute myeloid leukemia, *SCT* stem cell transplant

**Table 2:** List of attributes and levels for discrete choice experiment design

Attributes	Levels
Fever	Rarely/never
	Occasionally
	Frequently
Lack of energy	Occasionally
	Frequently
	All the time
Problems with daily function	Able to work normally
	Not able to work but able to perform daily activities
	Not able to perform daily activities but able to self-care (i.e., washing and dressing oneself)
	Not able to take care of self, bedridden most of the time
Anxious/depressed	Occasionally
	Frequently
Blood transfusions	Rarely/never
	Occasionally
	Frequently
Hospitalized	Rarely/never
	Occasionally
	Frequently
	All the time
Serious infection	Present
	Absent
Severe diarrhea	Present
	Absent
Severe redness/skin peeling	Present
	Absent
Abnormally low blood cell counts	Present
	Absent
Duration of life	1 year
	4 years
	7 years
	10 years

#### Choice card design

After the attributes and levels were determined, 144 choice cards with two hypothetical health scenarios on each card (see the Table 3 for an example) were constructed using the SAS macro package on DCE design to achieve an orthogonal, balanced, and efficient design, as suggested in the literature [18]. These choice cards were randomly divided into 12 groups with 12 choice cards per group. Each participant was randomly assigned to respond to one of the 12 groups and shown the 12 choice cards within the group in random order. In addition, two more choice cards were designed to assess internal validity (i.e. using the test-retest assessment) of the responses from the same participant. The two choice cards had the same pair of scenarios but in reverse order (i.e., scenario A in one choice card was scenario B in the other choice card) while one scenario was designed to be a dominant preferred option over the other scenario.

**Table 3:** Example discrete choice experiment choice card

Scenario features	Scenario A	Scenario B
A. Fever	Occasionally	Frequently
B. Lack of energy	Frequently	All the time
C. Problems with daily function	Not able to take care of self, bedridden most of the time	Able to work normally
D. Anxious/depressed	Occasionally	Frequently
E. Blood transfusions	Occasionally	Rarely/never
F. Hospitalized	All the time	Occasionally
G. Serious infection	**Present**	Absent
H. Severe diarrhea	Absent	**Present**
I. Severe redness/skin peeling	Absent	**Present**
J. Abnormally low blood cell counts	**Present**	Absent
K. Duration of life	4 years	1 year

#### Survey design

An online survey was designed to collect responses from a nationally representative sample of the US adult population. The survey consisted of three sections, including the collection of participant characteristics, a brief tutorial for completing DCE questions, and the set of DCE questions for participants to make preference decisions. Questions on participant characteristics included age, sex, education level, employment status, income level, marital status, comorbidities, self-reported general health status, and whether their family/friends have or had life-threatening conditions. The tutorial provided definitions of the attributes and levels associated with a life-threatening condition using layman language (Appendix 1) and a walk-through of elements to consider when making a decision based on the choice cards. Finally, each participant was presented with the series of DCE choice cards and asked to choose a preferred scenario between two options with varying levels of attributes on each card.

### Quantitative phase for utility valuation

#### Survey administration and participants

To ensure the clarity of the questionnaire and the choice cards, four individuals from the adult US general population with at least 6 years of formal education were recruited to pre-test the online survey separately. These individuals appreciated the seriousness of the life-threatening medical condition and thought the attributes and levels were well explained and easy for comprehension. Minor refinement to the wording for attributes and levels was made based on feedback from the pre-tests.

The questionnaire was developed and administered in English only. A total of 300 individuals representative of the adult general population of the US, in terms of age, sex, race, region, and income level, were recruited from an existing online panel of a market research firm to complete the finalized survey. Each participant was incentivized with $7 worth of panel points. The sample size was not determined by a formal power analysis partly because there was no specific hypothesis to test. Instead, the sample size was planned based on the recommendation in the literature [17]. The planned sample size also met the rule-of-thumb minimum sample size estimated using the formula $$ \frac{nta}{c}\ge 500 $$, where *c* was the largest number of levels for any one attribute, *t* was the number of tasks, and *a* was the number of alternatives per task [24]. Participants were required to be at least 18 years of age, have at least 6 years of formal education, and be willing to participate in the survey. The basic educational level was required in considering the somewhat complex task of making preference decisions based on choice cards. Anonymized data were collected through an online survey over the course of approximately 2 weeks in August 2016.

#### Statistical analyses

Characteristics of survey participants were summarized descriptively. Means with standard deviations (SD) were reported for continuous characteristics, and frequency counts and percentage were reported for categorical characteristics. The distribution of participants’ demographics was compared to those of the 2010 US Census to determine whether the sample was representative of the overall US adult population.

Responses to the preferences for hypothetical health scenarios were analyzed using a multivariable conditional logistic regression model with generalized estimating equations, where the dependent variable was participants’ preference decision on each choice card and the independent variables were the levels of attributes of the choice card [25, 26]. DCE studies assume that participants’ preference decisions are rational and depend on the attributes and levels given on a choice card, but are independent of their previous or future choices. As such, an independence correlation structure was used in the regression model to account for the correlations between multiple responses of the same participant. In addition, the regression model was estimated with two approaches: a main effect model and a model including duration of life as an interaction term with other attributes. The quasi-likelihood under the independence model criterion (QIC) was used to assess the model fit and determine the final model for utility score assessment [27]. The utility score of each health state was calculated using the regression coefficients from the final model for the levels of attributes that had predefined the health states (Table 1), as the product of exponentials of the corresponding coefficient estimates and the intercept. In DCE, the odds of selecting sicker health states were expected to be lower than selecting the relatively healthiest state of interest, CR. Because the level corresponding to the relatively healthiest category was used as the reference for each attribute in the regression model, coefficient estimates for other levels of the attributes were expected to be negative; the exponentials of the health states, where all attributes were considered, were expected to be constrained between 0 and 1. This approach projected the arbitrary scale of coefficient estimates from the conditional logistic regression to the utility scale between 0 and 1 to allow for the assigned utility values of 0 being death and 1 being perfect health. The disutility score of each SAE was calculated as the difference between the utility of CR and the product of the exponential of the corresponding SAE coefficient estimate and the intercept. The odds ratios (ORs) for levels of each attribute indicated the relative importance of the attribute level in participants’ preference decisions.

Analyses were conducted in SAS 9.4 (SAS Institute, Cary, NC). A *p* < 0.05 was used to determine statistical significance.

## Results

### Survey participant characteristics

The distribution of the 300 participants’ socio-demographics was within a 3% margin of the distribution reported in the 2010 US Census data [28], with the exception for education level, which is likely due to the participant eligibility criterion (Table 4). The mean (±SD) age of the 300 participants was 44.3 (±16.6) years and approximately half (51.3%) were female; 66.7% of participants were white, 37.0% were from the South, 59.7% were married or in a domestic partnership, 41.0% were full-time employees, and 20.7% reported a total household income greater than $100,000 for the past year. A proportion of 47.7% of participants had completed a bachelor’s degree or higher.

**Table 4:** Demographic Characteristics of Survey Participants

Characteristics	Total *N* = 300	2010 US Census^b^
Age (years), mean ± SD	44.3 ± 16.6	–
Age groups		
18 to 64 years	252 (84.0%)	82.8%
65 years and over	48 (16.0%)	17.2%
Female, N (%)	154 (51.3%)	51.5%
Race/ethnicity, N (%)		
American Indian or Alaskan Native	5 (1.7%)	0.7%
Asian	14 (4.7%)	4.7%
Black or African American	37 (12.3%)	12.2%
White/Caucasian	200 (66.7%)	63.7%
Hispanic, Latino, or of Spanish origin	40 (13.3%)	15.4%
Other^a^	3 (1.0%)	3.3%
Decline to answer	1 (0.3%)	–
US geographic region, N (%)		
Midwest	61 (20.3%)	21.7%
Northeast	58 (19.3%)	18.3%
South	111 (37.0%)	37.0%
West	70 (23.3%)	23.0%
Highest level of formal education, N (%)		
Completed elementary school, but not high school	9 (3.0%)	9.4%
Completed high school	65 (21.7%)	32.6%
Some college (did not complete college)	83 (27.7%)	29.4%
Completed bachelor’s degree or higher	143 (47.7%)	28.7%
Marital status, N (%)		
Single, never married	75 (25.0%)	26.9%
Married or in a domestic partnership	179 (59.7%)	56.5%
Widowed	10 (3.3%)	6.3%
Divorced	32 (10.7%)	10.4%
Separated	4 (1.3%)	2.4%
Employment status, N (%)		
Paid employee, full-time	123 (41.0%)	58.5% (employed)
Paid employee, part-time	26 (8.7%)	
Self-employed	11 (3.7%)	
Not working - unemployed/looking for work	23 (7.7%)	6.2%
Not working - disabled	18 (6.0%)	35.3% (not in labor force)
Not working - retired	59 (19.7%)	
Not working - home-maker	19 (6.3%)	
Student	21 (7.0%)	
Total household income for the past year, N (%)		
Less than $20,000	59 (19.7%)	18.9%
Between $20,000 and $29,999	34 (11.3%)	11.7%
Between $30,000 and $39,999	31 (10.3%)	10.6%
Between $40,000 and $49,999	24 (8.0%)	9.0%
Between $50,000 and $59,999	24 (8.0%)	8.0%
Between $60,000 and $74,999	28 (9.3%)	10.1%
Between $75,000 and $99,999	38 (12.7%)	11.5%
$100,000 or more	62 (20.7%)	20.2%

Abbreviations: *SD* standard deviation, *US* United States

^a^Other responses included Hawaiian and mixed race

^b^For the 2010 US Census data, the prevalence of age groups, sex, geographic regions, education levels, and marital status was estimated among persons 18 years of age and over. The prevalence of race/ethnicity was estimated among US population. The prevalence of employment status was estimated among persons 16 years of age and over. The prevalence of household income was estimated among the total number of households

More than half (60.7%) of the participants considered their health status to be very good or excellent, and only 2.7% rated their current health status as poor. The most commonly reported chronic comorbidity was anxiety or depression (28%), followed by hypertension (23.3%) and obesity (15.3%); 37.0% reported no chronic health conditions. Approximately half (51.3%) of the participants did not have a family member or close friend who had been diagnosed with a life-threatening illness, while 32.7%, 10.3%, and 22.0% of participants had a family member or close friend diagnosed with a solid tumor, blood cancer, or another life-threatening condition, respectively (Table 5).

**Table 5:** Health Characteristics of Survey Participants

Characteristics	*N* = 300
Self-assessed current health status, N (%)	
Excellent	69 (23.0%)
Very good	113 (37.7%)
Good	79 (26.3%)
Fair	31 (10.3%)
Poor	8 (2.7%)
Chronic comorbidities^a^, N (%)	
Anxiety / depression	84 (28.0%)
Cancer	19 (6.3%)
Cerebrovascular disease (e.g., stroke)	8 (2.7%)
Congestive heart failure	4 (1.3%)
Chronic kidney disease	4 (1.3%)
Diabetes (types I or II)	33 (11.0%)
Hypertension (high blood pressure)	70 (23.3%)
Hyperlipidemia	29 (9.7%)
Inflammatory bowel disease or other chronic bowel disease	13 (4.3%)
Ischemic heart disease (e.g., angina, myocardial infarction)	3 (1.0%)
Liver disease (e.g., chronic hepatitis)	5 (1.7%)
Lung disease (e.g., asthma or chronic obstructive pulmonary disease)	13 (4.3%)
Rheumatoid arthritis	20 (6.7%)
Obesity	46 (15.3%)
Other auto-immune diseases (e.g., psoriasis, thyroid disease, lupus)	18 (6.0%)
Other conditions	29 (9.7%)
No chronic health conditions	111 (37.0%)
Family member/close friend diagnosed with a life-threatening illness^a^, N (%)	
Diagnosed with solid tumor (e.g., breast, lung, or prostate cancer)	98 (32.7%)
Diagnosed with blood cancer (e.g., leukemia, lymphoma, myeloma)	31 (10.3%)
Diagnosed with other life-threatening condition (e.g., heart attack, stroke)	66 (22.0%)
No family member/close friend diagnosed with a life-threatening illness	154 (51.3%)

^a^Participants could select more than one option

Analysis of the responses to the choice cards assessing the internal validity of the participants’ responses showed 14.7% of the participants failed the test-retest validity test.

### Utility scores for treatment-related AML health states

Both the main effect model and the model including duration of life as an interaction term with other attributes were conducted. When including duration of life as an interaction term with other attributes, the model fit was worse than the main effect model (QIC: 4796.8 vs. 4439.2). As such, the results from the main effect model were used to calculate utility values based on the predefined health states. It was assumed that the residual factors differentiating CR from full health (utility = 1) were included in the intercept and the odds of selecting sicker health states were expected to be lower than the odds of selecting the relatively healthiest state of interest, CR.

Among all attributes, participants placed the highest value when having fewest problems with daily function, and the lowest value on energy levels. In comparison to not being able to take care of self, being able to work normally, to perform daily activities, and to self-care had large ORs (3.2, 2.4, and 1.8, respectively; all *p* < 0.001) (Table 6). This indicates, for example, that holding other attributes to be the same, the likelihood that a person would choose the scenario with being able to work normally was three times over the scenario with not being able to take care of self. Compared with being hospitalized all the time, being hospitalized rarely/never and being hospitalized frequently/occasionally were associated with ORs of 1.7 and 1.4, respectively (both *p* < 0.001). Similarly, less severe levels for other attributes were generally preferred over the most severe level (Table 6). To be noted, occasional fever or anxiety/depression was not significantly preferred over frequent occurrence; occasional or frequent lack of energy were not significantly preferred over lack of energy all the time. Among the four chemotherapy-related SAEs considered in this study, avoiding serious infection was valued highest (OR = 1.33; *p* < 0.001).

**Table 6:** Preferences for Features of AML Health States^a^

Features	Coefficient	OR	95% CI	*p*
Duration of life (years)	0.108	1.115	(1.09, 1.14)	< 0.001*
Fever				
Occasionally vs. *frequently*	0.022	1.022	(0.91, 1.15)	0.722
Rarely/never vs. *frequently*	0.235	1.265	(1.12, 1.43)	< 0.001*
Lack of energy				
Frequently vs. *all the time*	0.015	1.015	(0.90, 1.14)	0.795
Occasionally vs. *all the time*	0.098	1.103	(0.97, 1.26)	0.149
Problems with daily function				
Not being able to perform daily activities but able to self-care (i.e., washing and dressing oneself) vs. *not being able to take care of self, bedridden most of the time*	0.581	1.788	(1.50, 2.13)	< 0.001*
Not able to work but able to perform daily activities vs. *not being able to take care of self, bedridden most of the time*	0.887	2.428	(2.01, 2.93)	< 0.001*
Able to work normally vs. *not being able to take care of self, bedridden most of the time*	1.173	3.233	(2.64, 3.96)	< 0.001*
Anxious/depressed				
Occasionally vs. *frequently*	0.065	1.067	(0.96, 1.19)	0.242
Blood transfusion				
Occasionally vs. *frequently*	0.141	1.152	(1.02, 1.30)	0.025*
Rarely/never vs. *frequently*	0.167	1.181	(1.05, 1.33)	0.008*
Hospitalized				
Frequently/occasionally vs. *all the time*	0.347	1.415	(1.23, 1.63)	< 0.001*
Rarely/never vs. *all the time*	0.553	1.738	(1.46, 2.06)	< 0.001*
Avoiding risk of chemotherapy-related SAEs				
Serious infection	0.287	1.332	(1.21, 1.47)	< 0.001*
Severe diarrhea	0.225	1.252	(1.12, 1.39)	< 0.001*
Severe redness/skin peeling	0.071	1.073	(0.98, 1.18)	0.136
Abnormally low blood cell counts	0.121	1.129	(1.01, 1.26)	0.034*

*p < 0.05; Abbreviations: *SAEs* serious adverse events, *CI* confidence interval, *OR* odds ratio

^a^The estimated intercept in this model is − 0.134

The utility values of each health state are presented in Table 7. The utility score of each health state was calculated as the product of exponentials of the corresponding attribute coefficients and the intercept. For example, the utility score of “no complete remission” was *e*^−0.1340 − 0.2352 − 0.0830 − 0.5924 − 0.0648 − 0.0252 − 0.2057^ = 0.262. As would be expected, CR had the highest aggregated utility value (0.875) and a SCT event had the lowest value (0.158). The aggregated utility values of no CR, relapse, and the short-term period following SCT were 0.262, 0.355, and 0.398, respectively. Serious infection had the highest disutility (−0.218) and severe redness/skin peeling (−0.060) had the lowest disutility (Table 7). Sensitivity analysis was conducted when excluding those respondents who failed the test-retest validity test. The utility value differences between the sensitivity analysis and the full sample analysis varied between 0.015 and 0.057.

**Table 7:** Aggregated Utilities for AML Health States and Disutilities for Severe Adverse Events

Health State	Utility Values
Complete remission	0.875
No complete remission	0.262
Relapse	0.355
Stem cell transplant event	0.158
Post-stem cell transplant short term	0.398
Severe Adverse Events	Disutility from complete remission
Serious infection	−0.218
Severe diarrhea	−0.176
Severe redness/skin peeling	−0.060
Abnormally low blood counts	−0.100

Abbreviations: *AML* acute myeloid leukemia

## Discussion

AML is a rapidly progressive disease, and as a result, patients usually receive aggressive and often complex, treatments such as chemotherapies and SCT. These treatments may be life-saving, but can also negatively affect patient HRQL profoundly over both the short term and long term [4], particularly among older patients [5]. A 2004 literature review by Radaelli et al. assessed HRQL among patients with AML and found that HRQL score dropped shortly after diagnosis and persisted low throughout the course of therapy [4]. Furthermore, while long-term survivors regained HRQL nearly completely, in some cases, substantial negative impacts may persist well after treatment has ended. As such, clear definitions of health states for AML and proper utility valuations of these health states are necessary to reflect the benefits and risks associated with new therapies [29]. The availability of such values enables the incorporation of QALYs for cost-effectiveness/cost-utility analysis of these therapies. This study estimated the utility values for pre-defined, based on literature and consultation of clinical experts, treatment-related AML health states, and disutilities of treatment-related SAEs from a US societal perspective. A combination of the attributes, which considered patient-reported symptoms, function, mental health, and the care process, performed well in the descriptive system used in this study for AML health states categorization. Health states associated with a greater degree of independence and ability to function or work normally were highly preferred.

SG and iterative TTO are two frequently used methods to eliciting preference utility values. However, these methods usually involve in-person interviews and thus, most of the time data would be collected from a convenient sample from the general population when eliciting preferences. Moreover, the concept of SG or TTO are not always easily grasped by the respondents. Frequently, data from respondents who fail to understand those tasks are excluded from final analyses. The DCE method has attracted researchers in the healthcare field in recent years with its relatively simpler design and easier implementation than the traditional methods. Data from all respondents can be included for analysis and having a sample that is national representative is practical, as demonstrated in this study. In a comparative study, the health utility measurement scale, EQ-5D, was used to facilitate the utility estimation using both DCE and iterative TTO; the results of that study supported DCE to be a valid tool for this purpose [20, 21].

The current study is the first estimation of societal preferences using the DCE approach for disease-specific utilities, namely the AML treatment-related health states. Using the DCE approach, this study applied the statistical approach to constrain the upper and lower limit for utility estimation to be 0 and 1 to represent death and full health. The pre-defined health states utility estimation considered the intercept from the model to account for the residual factors not captured by the included attributes. This is because even when an AML patient is in remission, this patient is not likely in full health due to those residual factors. This approach is different from the one typically applied with EQ-5D, where a person who has no problem with any of the five dimensions of interest is assigned to have a value of 1 (full health). Admittedly, this definition of full health has been criticized for not considering a person’s general well-being [30–32]. On the other hand, this study arbitrarily constrained the lower bound of the utility value to be 0, meaning no health states worse than dead. Our study focused on estimating the utility values for the five pre-defined health states, which are believed to be preferred states to death. Therefore, this constraint is reasonable. Within the current attributes considered for the health states, a negative utility value cannot be derived. Cognizant a health state being worse than dead possible (e.g., in the EQ-5D value set), in extreme situations, an AML patient could have a negative utility value when considering the dis-utilities due to SAEs should be experienced.

Results from this study estimated the utility values for five health states associated with newly diagnosed patients with AML fit for standard chemotherapy. The utility values ranged from 0.875 for CR to 0.158 for SCT event. Several of the utility values estimated in this study were within the range of values reported previously for other hematologic neoplasms, regardless of methodology for assessment. For example, the utility value of CR for acute lymphoblastic leukemia (ALL) using TTO was 0.86 [33] and for chronic lymphoblastic leukemia (CLL) using SG was 0.91 [34] in the UK general population; the utility value of CR for chronic myelogenous leukemia (CML) was 0.84 using the TTO approach taking responses from convenience samples in Canada, the US, the UK, and Australia [35]. The study for CML included a similar health state of “not responding to treatment” with a utility value of 0.21 [35], similar to our “no CR” health state with a utility value of 0.262. In addition to similarities in utility values associated with CR and no CR, we also found similar values from these studies when evaluating safety. For example, the study for CLL utility assessment also included grade 3/4 pneumonia and found a disutility of − 0.20 [34]. This value is also similar to the finding of − 0.218 for grade 3/4 serious infection in our study.

Because of a relatively small number of patients affected, therapy development for rare diseases has unique challenges, particularly when a large sample size is needed for assessing relevant outcomes. AML in particular has posed its own challenge due to the presence of many subtypes with various gene mutations [14]. For example, only 30% of adults with newly diagnosed AML have mutations in the fms-related tyrosine kinase 3 gene (FLT3) which is associated with a high relapse rate and poor prognosis. The findings from this study support the use of DCE methodology as a valid approach for obtaining societal preferences for disease-specific treatment-related health states. This can be a particularly valuable tool for generating utility values for rare diseases such as AML. The findings of this study may be generalizable for AML, given that the utility values were estimated from the societal perspective, with a national representative sample in the US. The preference values estimated in this study can be applied in economic evaluations of AML interventions.

The findings from this study may also have implications in addressing the unmet needs when developing new therapies for AML. The results showed that the most preferred attributes were those involving the maintenance of independent functioning and avoidance of hospitalization. CR had the highest utility value (0.875), followed by post-SCT short-term recovery (0.398), relapse (0.355), no CR (0.262), and SCT (0.158). These values reflect the extent to which the society place on treatment-related AML health states though it is important to keep in mind that SCT remains the only potentially curative treatment for AML. The relatively low utility value of SCT may mainly be a reflection of the restriction associated with the procedure that people strongly preferred to avoid (e.g., hospitalized with frequent blood transfusion). Admittedly, the preferred attributes resulting from this study were from the perspective of the general population. For future AML therapy development, the preferences coming from patents themselves should also be taken into account.

Available AML therapies with curative intent have remained constant for several decades, although emerging therapies may change the treatment landscape [36, 37]. Utility values and societal preferences, such as those produced by this study, can aid in economic assessments of new therapies for AML as well as identify unmet needs in the patient population. Future research on approaches to reduce the negative impact of AML treatment, with the goals of maintaining independence and minimizing life disruption as well as eliciting disease remission, would be valuable for the future.

### Limitations

The online panel-based survey was answered by adults from the general population in the US, and the utility values were generated from a societal perspective. Participant eligibility for the survey was determined based on self-reported information. Overall, the participants reflected the demographics of the US population, although by study design, they had a higher educational attainment (47.7% with a bachelor’s degree or higher) than the general population (33%) [38]. However, the preferences of patients with AML, and according utilities, may differ from those of the general US population and those of other countries. For example, in this study, we observed that the likelihood of participants choosing the scenario of being able to work normally was over 3 times that of choosing a scenario involving not being able to take care of oneself. When considering that the median age of AML diagnosis is 68, the study participants’ preference for ability to work vs. the ability to take care of themselves could be different from that of the general population.

Among all participants, 85% provided consistent responses on the choice cards for test and retest validity, suggesting that the choice cards were well understood and that participants made reasonable preference decisions [39]. AML is a complex disease, which has heterogeneous course and therapeutic options. As such, AML patients could have more health states based on the given treatments and stage of disease. Our study included five health states for patients newly diagnosed with AML who are fit for standard induction chemotherapy and for whom SCT is a potentially curative therapy. However, the DCE design provides flexibility in deriving utility values for additional health states. For example, the CR health state was currently defined for *CR after all treatments are completed*. By applying different levels of the attributes, *CR during consolidation* may be defined with fever (occasionally), lack of energy (occasionally), problems with daily function (not able to work but able to perform daily activities), anxious/depressed (occasionally), blood transfusion (occasionally), and hospitalized (occasionally), and thus have an estimated utility value of 0.421.

It should be noted that the treatment-related AML health states and the attributes/levels used to define these health states were based on literature review and experts’ input. No separate patient focus groups were conducted to confirm the content validity and attributes’ relevance to health states. Ideally, focus groups should be conducted to include patients across the proposed health states and with some patients who have experienced most of the health states. However, due to the rarity of the disease, the age of the patients, and the frailty of the patients in certain health states, it was not practical to carry out focus groups among this particular patient population. We believe the current two approaches together used to determine health states, particularly the rounds of discussions with the experts, should have reflected the common health states that clinicians observe in their daily clinical practice. Of course, future studies applying the methodology in this research should consider including patient focus groups when possible.

## Conclusion

AML and its associated treatments can be associated with reduced HRQL and impaired ability to perform daily activities. The level of impairment depends on health status, including SAEs experienced during treatment. This analysis of societal preferences for treatment-related AML health states showed a strong preference for maintaining independence and avoidance of hospitalization. The utility values of treatment-related AML health states and disutilities of SAEs generated in the present study could be used to aid in economic analyses of AML treatments. The results of this study support the use of DCE methodology as a valid approach to obtain societal preference values for treatment-related health states.

## Appendix 1

### Definitions of the attributes associated with a life-threatening condition

- **Fever**: The life-threatening condition impairs your immune system and increases your chance of developing a fever, an abnormally high body temperature often accompanied by shivering and headaches. Severe fevers will require medical attention and, in some cases, a patient will need to be admitted to a hospital to receive treatment for the fever.
- **Lack of energy:** The life-threatening condition may cause fatigue characterized by extreme tiredness, even after sufficent rest and in the absence of any physical exertion.
- **Problems with daily function:** The life-threatening condition can impact ability to perform daily activities such as work, chores, self-care (i.e., washing and dressing oneself), and family or leisure activities. In particularly severe cases, a patient may be bedridden most of the time.
- **Anxious/depressed:** The life-threatening condition may cause considerable emotional stress including persistent feelings of sadness, anxiety, depression, or fear.
- **Duration of life:** The life-threatening condition will impact life expectancy. The disease-related scenarios that you are presented with will differ in the number of years you would survive with the condition.
- **Blood transfusions at a healthcare facility:** The life-threatening condition can cause abnormally low blood cell counts that may necesitate blood tranfusions, a medical procedure where donated blood is added to a patient’s blood through an intravenous (IV) line in order to boost blood cell counts that are too low. Blood transfusions usually take one to 4 hours. The frequency of blood transfusions will vary depending on the patient’s body’s ability to produce blood adequately.
- **Hospitalization:** The life-threatening condition may require a patient to be admitted to a hospital to receive intentsive treatment or be closely monitored. The frequency and duration of these hospitalizations will vary depending upon the severity of the patient’s condition and the types of therapy required to treat the condition.
- **Serious infection:** A patient could be at risk for several kinds of infections because of treatment for the life-threatening condition. For example, infections could occur in the lungs (e.g., pneumonia), in the blood, or in the skin (e.g., injection site-related). Severe infections are medical emergencies requiring admission to a hospital to receive immediate antibiotic treatment. Without timely treatment, in extreme cases, these infections could lead to death.
- **Severe diarrhea:** This treatment-related problem is characterized by frequent and watery bowel movements. It is frequently associated with abdominal pain or cramping, which could further impact ability to perform daily activities. Severe diarrhea will require medical attention with fluid support and possibly antibiotics. In some cases, a patient needs to be admitted to a hospital to get treated for severe diarrhea.
- **Severe redness/skin peeling:** This treatment-related problem is a severe skin disorder characterized by itchiness, redness, and visible peeling of the skin in part of the body or, in some cases, the whole body. Without timely treatment, a patient may need to be hospitalized in order to control the skin problem. Treatments include antihistamines and creams, and corticosteroids for severe cases.
- **Abnormally low blood cell counts:** A patient could be at risk for abnormally low blood cell counts such as red blood cells, white blood cells, and platelets. Having abnormally low blood cell counts may lead to fatigue, dizziness, headaches, increased risk for bruising/bleeding, fever, and infections. Without timely treatment, it may lead to hospitalization, even death.

## Abbreviations

AE: Adverse event
ALL: Acute lymphoblastic leukemia
AML: Acute myeloid leukemia
CLL: Chronic lymphoblastic leukemia
CML: Chronic myelogenous leukemia
CR: Complete remission
DCE: Discrete choice experiment
FLT3: Fms-related tyrosine kinase 3 gene
HRQL: Health-related quality of life
OR: Odds ratio
QIC: Quasi-likelihood under the independence model criterion
SAE: Severe adverse event
SCT: Stem cell transplant
SD: Standard deviations
SG: Standard gamble
TTO: Iterative time-trade-off
US: United States
